# Case report: Reoperative parathyroidectomy for large ectopic hyperplastic parathyroid in the mediastinum of a patient with recurrent secondary hyperparathyroidism

**DOI:** 10.3389/fsurg.2022.921026

**Published:** 2022-07-27

**Authors:** Yong Lv, Qiuyuan Wang, Ling Zhang, Qing Zhou, Zhiyu Mi, Yi Wu, Jingning Cheng

**Affiliations:** ^1^ Department of Otolaryngology-Head and Neck Surgery, China-Japan Friendship Hospital, Beijing, China; ^2^ Department of Orthopaedic Surgery, China-Japan Friendship Hospital, Beijing, China; ^3^ Graduate School of Beijing University of Chinese Medicine, Beijing, China; ^4^ Department of Nephrology, China-Japan Friendship Hospital, Beijing, China

**Keywords:** reoperative parathyroidectomy, secondary hyperparathyroidism, chronic renal failure (CRF), hemodialysis, hypocalcemia, IPTH

## Abstract

**Introduction:**

Secondary hyperparathyroidism (SHPT) is a common complication in hemodialysis patients with chronic renal failure uremia. For severe SHPT, parathyroidectomy is effective. Owing to the variability in parathyroid anatomy, surgical parathyroidectomy can be complex and many patients experience recurrent SHPT, which may require repeated surgery. These cases pose significant challenges to surgeons.

**Case description:**

An elderly woman with recurrent severe SHPT was admitted to our hospital. Preoperative methoxyisobutylisonitrile (MIBI) examination found a large ectopic parathyroid gland in the superior mediastinum, and she underwent reoperative parathyroidectomy. A large parathyroid gland in the right anterior mediastinum and another parathyroid gland in the left lingual lobe of the thymus were removed. The patient had postoperative hypocalcemia that was successfully corrected with calcium supplementation *via* femoral vein catheterization. During the 1-year postoperative follow-up, the patient's iPTH was well controlled and her blood calcium was within the normal range.

**Conclusion:**

We report a case of parathyroidectomy to remove multifocal ectopic hyperplastic parathyroid tissue in the mediastinum. Preoperative MIBI accurately detected the lesions. Calcium supplementation *via* femoral vein catheterization successfully corrected postoperative hypocalcemia. Postoperative follow-up for 1 year indicated that the surgery was successful.

## Introduction

Secondary hyperparathyroidism (SHPT) is a common complication in hemodialysis patients with chronic renal failure uremia (1). Chronic renal failure leads to long-term dysregulation of calcium–phosphorus metabolism, a compensatory increase in parathyroid hormone (PTH) secretion, and parathyroid hyperplasia. Drug therapy can be used to treat early-stage SHPT (2, 3). Calcimimetic is a popular treatment choice for managing SHPT patients (4, 5). However, for severe SHPT and patients with drug resistance, surgical treatment is required (6–8). This article presents a complex reoperative case of parathyroidectomy for multifocal large ectopic hyperplastic parathyroid tissue in a hemodialysis patient with recurrent SHPT.

## Case description

### Patient history

A 69-year-old woman was admitted to our hospital for persistent bone pain, weakness, and skin pruritus. She had been diagnosed with chronic renal insufficiency more than 30 years prior to her presentation. Twelve years prior to the presentation, she began regular hemodialysis three times per week. Five years before the presentation, she began to experience symptoms of bone pain, weakness, and skin pruritus. She visited a local hospital, and the clinicians found an elevated iPTH (above 2,000 pg/ml). The patient then received parathyroidectomy surgery in that hospital, during which three parathyroid glands were identified and removed intraoperatively (according to the patient's statement; surgical notes were not provided). Unfortunately, although her iPTH decreased postoperatively, it remained higher than normal (up to 1,000 pg/ml). The patient took Rocaltrol after surgery, but her iPTH level did not decrease; in contrast, it gradually increased. After 1 year of surgery, the highest iPTH level was 3,000 mg/ml. The patient continued to take Rocaltrol, but her iPTH level continued to worsen. One month before admission to our hospital, her iPTH was up to 4,000 pg/ml according to laboratory tests performed at her local hospital.

### Diagnostic assessment

**Figure 1:**
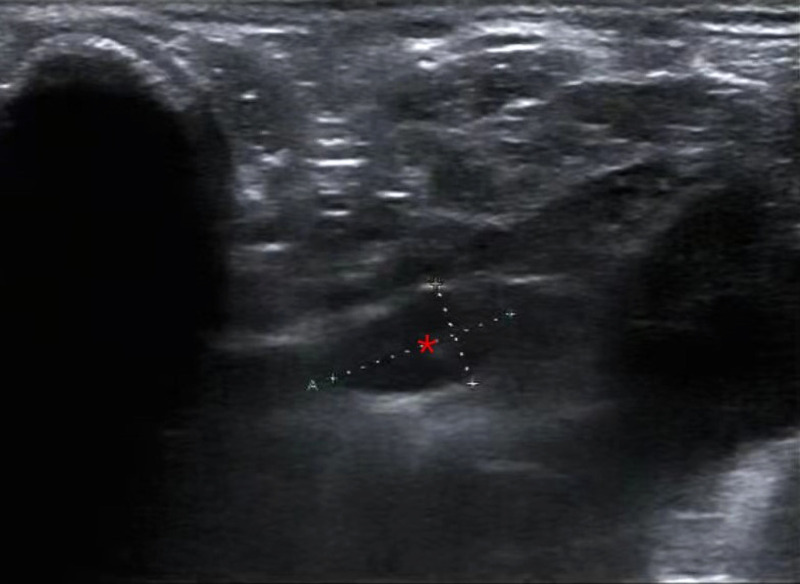
Parathyroid ultrasound showing a hypoechoic nodule (*) on the left side of the suprasternal fossa.

**Figure 2:**
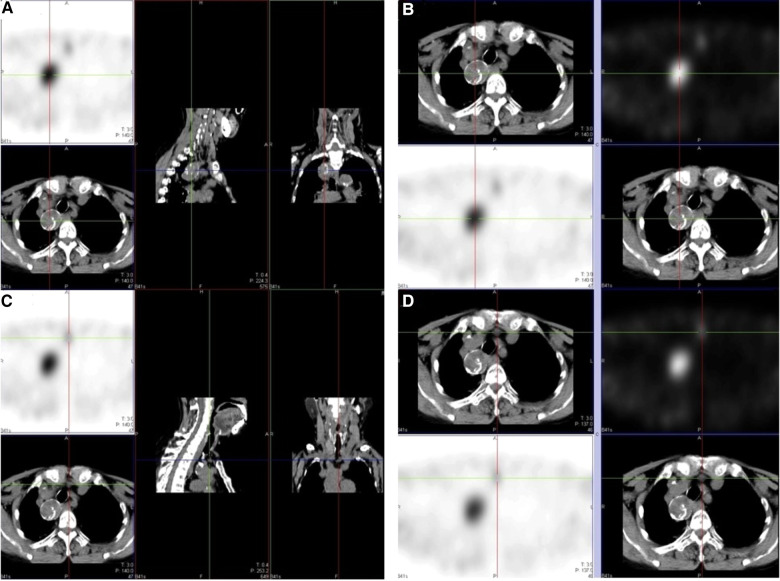
MIBI examination. (**A, B**) Large soft tissue density nodule accompanied by eggshell calcification in the superior mediastinum, located behind the innominate artery and beside the trachea; (**C, D**) soft tissue density nodule in the suprasternal fossa.

After admission to our hospital, a parathyroid ultrasound indicated a hypoechoic nodule on the left side of the suprasternal fossa (Figure 1). Methoxyisobutylisonitrile (MIBI) examination showed a 3.2 cm × 3.0 cm soft tissue density nodule accompanied by eggshell calcification in the superior mediastinum, located behind the innominate artery and beside the trachea (Figures 2A, B). MIBI also showed an approximately 1.2 cm × 1.0 cm soft tissue density nodule in the suprasternal fossa (Figures 2C, D). The MIBI scans of the two foci were all high uptake. Preoperative blood biochemistry analysis showed that the iPTH was above 3,000 pg/ml, calcium was 2.24 mmol/L, phosphorus was 1.46 mmol/L, and alkaline phosphatase (ALP) was 452 IU/L.

### Therapeutic interventions

**Figure 3:**
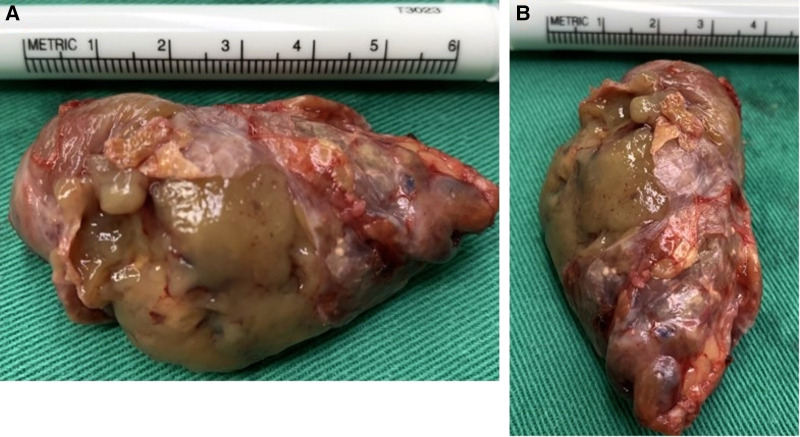
Large parathyroid in the right anterior mediastinum after removal. (**A**) Length of the mass was approximately 7 cm; (**B**) width was approximately 3.5 cm.

Bedside hemodialysis was arranged 1 day prior to the planned parathyroidectomy surgery. Under general anesthesia, the patient underwent a second parathyroidectomy *via* an anterior transcervical low-neck incision. After routine isolation through the subcutaneous tissue and adhered scar tissue, structures such as the trachea in the root of the neck, the cervical transverse arteries and veins, and the thymic tongue lobe were preserved. Taking particular care to protect the blood vessels of the root of the neck, we advanced a probe along the right side of the trachea to the upper mediastinum. The mass was probed near the trachea, above the aortic arch. The blood vessels around the mass were carefully separated and transected by electrocoagulation hemostasis. We gradually separated the mass from surrounding tissue and protected the capsule so it could remain as intact as possible during the parathyroidectomy. However, due to the large size of the mass, the capsule broke during removal. We removed the lump intact, and no macroscopically visible ectopic parathyroid tissue remained. The surgical site was irrigated with copious amounts of normal saline to prevent implantation of any exfoliated tumor cells. Intraoperatively, we also removed a very large parathyroid gland in the right anterior mediastinum. This second mass was 7 cm × 3.5 cm in size and weighed 23.72 g (Figures 3A, B). We also removed another parathyroid gland in the left lingual lobe of the thymus. The diameter of the third mass was 1 cm, and its weight was 0.69 g. No injury was found when exploring the bilateral recurrent laryngeal nerve. Furthermore, we used a nerve monitor to measure the electrical potential of the recurrent laryngeal nerve (V2, R2) and found no significant change before and after removing the parathyroids. Finally, we placed a drainage tube, sutured the soft tissue, and closed the wound.

**Figure 4:**
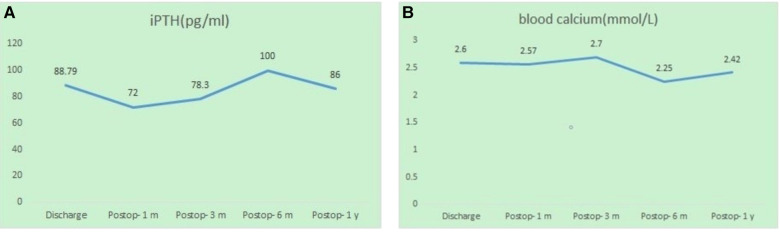
Results of the 1-year follow-up. (**A**) iPTH level; (**B**) blood calcium level.

The patient returned to the ward safely after recovery from anesthesia. An immediate postoperative blood test showed that iPTH had decreased to 102.7 pg/ml. Postoperatively, the patient reported that her symptoms of bone pain, weakness, and skin pruritus were significantly reduced. One day after surgery, the patient underwent bedside hemodialysis. The postoperative blood electrolyte analysis indicated hypocalcemia (blood calcium was 1.69 mmol/L). The patient felt numbness and acanthesthesia in her fingers. We treated her with 30 g intravenous calcium gluconate diluted in 300 ml of 0.9% NS *via* femoral vein catheterization. On the second day of postsurgery, the patient's blood calcium recovered to 2.6 mmol/L and the iPTH decreased to 88.79 pg/ml. The numbness and acanthesthesia in her fingers also disappeared, so we stopped the intravenous calcium treatment. The patient was discharged from our hospital on the third day post-surgery and continued oral calcium and calcitriol supplementation (calcium carbonate 1 g TID and calcitriol 2 g BID). During 1-year follow-up, the patient's iPTH was well controlled and the blood calcium remained within normal limits (Figure 4).

## Discussion

Regarding surgical indication, the patient had received regular hemodialysis for 12 years and had developed SHPT at least 5 years prior to presentation. Although she had previously undergone a parathyroidectomy, her postoperative iPTH had remained elevated (1,000 pg/ml) and gradually increased over time. This indicated the continued presence of parathyroid tissue with excessive parathyroid hormone secretion, suggesting incomplete removal of parathyroid tissue in the primary surgery. The maximum iPTH level of this patient was above 4,000 pg/ml. Such a high iPTH level can significantly exacerbate the dysregulated mineral and bone metabolism associated with chronic renal insufficiency and further affect the cardiovascular system, as well as the central and peripheral nervous system (9–12). Drug therapy alone could not control the worsening parathyroid hyperplasia and the severe clinical symptoms in our patient. Therefore, reoperative parathyroidectomy was the best choice for this patient.

Accurate imaging is crucial for successful reoperative parathyroidectomy (14, 15). This case highlights the diagnostic value of the MIBI examination for ectopic parathyroid detection. In this case, the initial parathyroid ultrasound examination did not find the large ectopic parathyroid in the superior mediastinum, but the MIBI did. Although the benefits that MIBI provides in cases of parathyroidectomy for SHPT are still controversial (14, 16–19), we suggest that for complex SHPT cases, especially reoperative cases, MIBI examination can help clinicians avoid missed diagnoses and improve preoperative planning.

As a reoperative parathyroidectomy surgery, the main goal, in this case, was to find and remove all remaining parathyroid tissue (13). This also became the biggest challenge during the surgery. The preoperative MIBI found a large ectopic parathyroid located deep in the superior mediastinum. This location made it challenging to expose the target lesions through the common anterior transcervical approach. Excellent surgical skills and familiarity with anatomy were required for the surgeons to complete the surgery successfully. Intraoperatively, it was necessary to pay close attention to blood vessel protection and operate as bloodlessly as possible because once mediastinal hemorrhage occurs, hemostasis is very difficult. These aspects all required a higher standard of delicate surgical skills.

Postoperative management is also crucial for SHPT patients (20, 21). This case shows that clinicians need to closely monitor patients' blood calcium to promptly discover and treat hypocalcemia (6, 22). In recent years, the mode of multidisciplinary team (MDT) management has been introduced into the treatment of SHPT patients (23, 24). Since SHPT treatment is a long-term project, patient management should be both whole-process and comprehensive (20, 25). Postoperative follow-up is an important component of patient care.

## Conclusion

We report a case of reoperative parathyroidectomy for multifocal large ectopic hyperplastic parathyroid tissue within the mediastinum of a patient with recurrent SHPT. Preoperative MIBI accurately detected the lesions. Calcium supplementation *via* femoral vein catheterization successfully corrected postoperative hypocalcemia. One-year postoperative follow-up showed that the surgery was successful as the patient's iPTH remained well controlled and her blood calcium remained within the normal range.

## Data Availability

The original contributions presented in the study are included in the article/Supplementary Material; further inquiries can be directed to the corresponding author/s.
